# Impact of caregiver interventions on caregiver burden in adult traumatic brain injury: a systematic review and meta-analysis

**DOI:** 10.3389/fpubh.2025.1698592

**Published:** 2025-12-03

**Authors:** Chung Yan Ting, Zubair Ahmed

**Affiliations:** 1 Department of Inflammation and Ageing, School of Infection, Inflammation and Immunology, College of Medicine and Health, University of Birmingham, Birmingham, United Kingdom; 2 University Hospitals Birmingham NHS Foundations Trust, Birmingham, United Kingdom; 3 Centre for Trauma Sciences Research, University of Birmingham, Birmingham, United Kingdom; 4 Institute for Mental Health, University of Birmingham, Birmingham, United Kingdom

**Keywords:** caregiver interventions, caregiver programs, traumatic brain injury, caregiver burden, psychological distress, rehabilitation

## Abstract

**Background:**

Traumatic brain injury (TBI) has been known to cause physical and psychological dysfunction among patients. Most family members face numerous physical and psychological difficulties in caregiving, yet effective interventions remain limited. This study aims to (a) identify the effects of caregiver interventions on caregiver burden for traumatic brain injury patients, (b) evaluate the effects of these programs on caregivers' psychological distress.

**Methods:**

A systematic search was conducted in Pubmed, Medline and PsycINFO to search for randomized control trials (RCTs) that report the effects of caregiver interventions on care partners of TBI patients above the age of 18. The primary outcome was caregiver burden, which was measured mainly using the Zarit Burden Interview (ZBI). The secondary outcome was psychological distress, with the Brief Symptom Inventory-18 (BSI-18) as the main assessment used. The RoB-2 tool was used to assess the risk of bias.

**Results:**

Thirteen RCTs were identified after screening. Meta-analysis of the primary outcome (ZBI) showed significant improvements (*p* < 0.05) favoring intervention despite high heterogeneity in the 5 studies. Meta-analysis of the secondary outcome (BSI-18) indicated smaller but significant improvement (*p* = 0.02) with low heterogeneity in 3 studies.

**Conclusion:**

Results suggest that caregiver interventions are effective in improving caregiver burden and psychological distress in the future. However, due to high risk of bias in studies, the conclusion should be interpreted with caution before clinical application. Higher quality research should be conducted to ensure the effectiveness of caregiver programs.

## 1 Introduction

Traumatic brain injury (TBI) is one of the most common causes of trauma worldwide, affecting 20.8 million people worldwide (1). It causes physical, cognitive, functional and psychological difficulties among individuals with TBI, which makes caregiving challenging as it takes on the form of a chronic disease (2). About 70% of familial caregivers experience significant caregiver burden in the various populations, as most informal caregivers, i.e. family members including a spouse or a parent, are not ready to take up the role of caregiving after trauma hits (3). Studies have shown that caregivers encounter daily struggles in balancing their own needs and those of the person with TBI (4). They often experience psychological distress, changes in daily routines and relationships, physical strain, financial difficulties and shortage of resources, especially for moderate to severe TBI patients (5). Feeling overburdened by daily responsibilities is commonly reported among caregivers, with up to 20% caregivers experiencing significant depressive and somatic symptoms clinically (6). Social isolation also becomes a major challenge as social support is reduced (4). Substantial financial burden adds to caregivers' psychological stress as well (7). Although multiple factors contribute to caregiver burden, resilience could be built as long as caregivers have the perception that their needs are being met (8). Therefore, various caregiver interventions have been developed over the years to offer support to TBI caregivers in different stages of caregiving.

Conceptualisations of caregiver interventions have evolved from a predominantly problem-solving approach to a more strengths based approach during the 1990s to 2010s. Intervention content fell into two main categories: caregiving skills for supporting individuals with TBI and strategies for maintaining caregivers' health and well-being. Early sessions often focused on recognizing the symptoms of TBI, addressing cognitive and behavioral challenges and enhancing communication with the patient. A consistent finding across the literature was that effective programs combined educational information with opportunities for reflection and sharing in supportive environments. Thus structured programs that incorporate both practical skill building and emotional support were found to yield the most positive results (9). In recent decades, caregiver interventions for TBI have evolved from face-to face sessions to include a range of online methods of learning. Reported modalities include mobile health applications with online chat functions, telephone consultations, online problem solving interventions and video support groups aimed at improving accessibility for caregivers in remote areas (10, 11). While there was some controversy regarding the comparability of online and in-person interventions, studies have proved that online interventions can achieve equivalent outcomes when tailored to specific needs (12). For example, the Caregiver Wellness after Traumatic Brain Injury Program (CG-Well) has incorporated interactive games, inspirational quotes, supportive videos and photographs as creative methods of offering emotional support (13). Moreover, flexibility appears to be a recurrent advantage, allowing users to access the content at their own pace (14, 15).

In recent years, researchers have proposed several recommendations for designing personalized programs for caregivers of individuals with TBI, one of which emphasizes the principle of “respecting cultural differences” (16). An increasing trend of TBI caregiver interventions has been spotted over the last few years in middle income countries, such as Mexico, India and Indonesia (10, 17, 18). Another pilot study for Latino American caregivers was conducted to tailor to their needs, improving somatic symptoms and caregiver burden (19). These differences in cultural values can significantly influence how families engage with individuals living with TBI. Although numerous studies have examined the needs of TBI caregivers, there appears to be a notable gap in literature since 2018, when the most recent systematic review was published (20). The review identified methodological limitations in the existing evidence, including the need for more rigorous study designs and the adoption of outcome measures with higher validity and reliability. A separate systematic review on online TBI caregiver interventions further stated that small sample sizes limit the overall generalizability of results (12). Since 2018, several higher quality studies have been published, including interventions targeting different ethnic minorities and employing a range of delivery methods. Hence, the emerging body of research warrants comprehensive analysis to inform clinical thinking and guide the development of rehabilitation programs.

At present, no consensus exists on general guidelines for TBI caregiver interventions, underscoring the need to determine which program characteristics yield the most beneficial outcomes. The aim of this study is to evaluate the effectiveness of TBI caregiver interventions in alleviating caregiver burden and psychological distress through a systematic review and meta-analysis. Intervention effectiveness will be compared against standard hospital care to examine whether recently published randomized controlled trials differ from results in earlier reviews. Caregivers for adult TBI patients were included in the systematic review, excluding those caring for children and adolescents. The primary outcome of this study is caregiver burden while the secondary outcome includes measures of psychological distress, indicating symptoms of depression and anxiety. Both outcome measures were considered to determine whether caregiver programs effectively bring about positive changes in caregiver burden and stress.

## 2 Methods

### 2.1 Search strategy

The systematic review was conducted according to the Preferred Reporting of Items for Systematic Reviews and Meta-analysis (PRISMA) guidelines (21). The literature search was completed on 22nd April 2025 in three databases, namely Pubmed, MEDLINE and PsycINFO. For search terms, (“traumatic brain injury”) AND (“caregiver intervention” OR “caregiver program” OR “Caregiver” OR “family” OR “Dyad”) AND (“randomized controlled trial” OR “RCT”) were used. Gray literature was also sourced from searches in ProQuest Dissertations and Theses and OpenGrey databases, conference proceedings as well as from scanning the references in previous systematic reviews that included studies of TBI caregiver interventions (20, 22) (Table 1).

**Table 1:** Search terms used to identify relevant studies.

**Boolean search criteria:**
“Traumatic brain injury”
“Caregiver intervention” OR “Caregiver program” OR “Caregiver” OR “family” OR “Dyad”
“Randomized controlled trial” OR “RCT”

### 2.2 Inclusion and exclusion criteria

Studies were included if the following criteria were met: (1) written in English; (2) the study design is an RCT; (3) described an intervention for TBI caregivers or a program involving both TBI survivors and caregivers, with comparison to a control group; (4) enrolled adult patients as participants; (5) full text was available.

Exclusion criteria of the studies included the following: (1) did not involve caregivers in the study; (2) enrolled adolescents or children as participants (<18 years old); (3) included other brain injury patients as participants; (4) program involving caregivers of adolescents/ children with TBI (<18 years old); (5) full text was unavailable; (6) not an RCT.

Studies involving caregivers of adolescents/children with TBI were excluded as their needs differ from that of adult TBI patients due to anatomical and physiological differences of the brain (23). Papers with studies were not filtered by date to include all studies up to April 2025.

### 2.3 Data collection

A manual search was conducted on each database, where titles and abstracts were screened to identify suitable articles. Covidence software was used to identify duplicates and track the process of screening (24). Studies were then sought for full text analysis according to the inclusion and exclusion criteria. The initial screening was done by the primary author (CYT) and then reviewed by the co-author (ZA). Any discrepancies were discussed between the two authors and resolved through mutual agreement.

### 2.4 Data extraction

Data was extracted by the primary author (CYT) to a spreadsheet for further analysis. The data was then reviewed by the co-author (ZA) for verification. The characteristics of the studies, characteristics of participants, characteristics of intervention and control groups and their respective outcome measures were extracted (Table 2).

**Table 2:** Characteristics of the included studies.

**Authors**	**Country**	**Number of Participants**	**Age (mean)**	**Gender**	**Intervention programme**	**Control**	**Setting**	**Outcome measures**
Carlozzi et al. (14)	United States	Total: 254 Intervention: 126 Control: 128	52	Female: 79.1% Male: 20.9%	The CareQOL app, a mobile health app designed to promote care partner self-awareness (self monitoring) and self-care (push notifications)	Self-monitoring alone from CareQOL app	Patients' homes	Supervision Rating Scale, several PROMIS-CareQOL short forms, Mayo Portland Adaptability Inventory-Fourth Edition, the Posttraumatic Stress Disorder Checklist for DSM-5, COVID-19 HRQOL measure, modified version of the Caregiver Appraisal Scale
Ganefianty et al. (10)	Indonesia	Total: 74 Intervention: 37 Control: 37	43.8	Female: 83.8% Male: 16.2%	Education and face to face information meetings before discharge, online chat feature with weekly monitoring in health application after discharge	Standard care program in hospital	Hospital and patients' homes	Modified version of Caregiver Stress Self-assessment, Short form ZBI, readmission rate of patients with TBI
Hanks et al. (26)	United States	Total: 62 Intervention: 31 Control: 31	51	Female: 44.5 % Male: 55.5%	Weekly/ biweekly contact by trained mentors	No mentoring	Community	Peer Mentoring Questionnaire, BSI-18; Family Assessment Device, Coping Inventory for Stressful Situations; Short Michigan Alcohol Screening Test, Medical Outcomes Study 12-Item Short Form Health Survey and Community Integration Measure.
Løvstad et al. (29)	Norway	Total: 73 Intervention: 39 Control: 34	49 (median)	Not specified	Home-based intervention: six home visits and two telephone calls	Usual healthcare and rehabilitation services	Patients' homes	Caregiver Burden Scale (ZBI), Patients Health Questionnaire-9, EuroQol 5 Dimensions visual analog scale, Patient Competency Rating Scale Relative Form, Target Outcomes or main TBI-related problem areas from semi structured interview
McLaughlin et al. (15)	United States	Total: 201 Intervention: 104 Control: 97	48% in 36–50 age group	Female: 86.1% Male: 13.9 %	The Brain Injury Partners program, an interactive Website focused on advocacy, communication skills, and resources for families affected by brain injury	The BIAUSA Website, a website focusing on advocating TBI legislative issues	Community	Knowledge measures included 18 questions about the key learning objectives related to communication skills, Satisfaction with Life Scale, 17 VSTs for assessing skill application and behavioral intention
Moriaty et al. (27)	United States	Total: 81 Intervention: 40 Control: 41	41.6	Female: 93.8% Male: 6.2%	6 home visits and 2 telephone calls delivered by occupational therapists	Standard outpatient clinic-based TBI care by the multidisciplinary care team	Patients' homes	Center for Epidemiologic Studies Depression Scale, Modified Caregiver Appraisal Scale, Caregiver Relationship Satisfaction subscale
Niemeier et al. (9)	United States	Total: 93 Intervention: 42 Control: 51	51.3	Female: 82% Male: 18%	5-session manualized intervention with educational, stress and anxiety self-management, coping, and emotional support components.	5 packets of educational materials given without verbal explanation	Inpatient brain injury rehabilitation unit	Family Needs Questionnaire-Revised, knowledge assessment, ZBI and BSI-18
Perrin et al. (18)	Mexico, Colombia	Total: 89 Intervention: 44 Control: 45	41.9	Female: 80.9% Male: 19.1 %	A 1-hour face-to-face intervention session before the patient's hospital discharge, and four 1-hour in-home visits at 1, 2, 4, and 6 weeks after discharge	Standard care provided by the hospital	Hospital and patients' homes	Patient Health Questionnaire-9, ZBI, Self-Perceived Burden Scale
Powell et al. (11)	United States	Total: 153 Intervention: 77 Control: 76	49.7	Female: 82% Male: 18%	Individualized education and mentored problem-solving intervention focused on caregivers' primary concerns delivered via up to 10 telephone calls at 2-week intervals	Usual care	Community	Composite of Bakas Caregiving Outcomes Scale and BSI-18, Brief Coping with Problems Experienced Questionnaire
Rasmussen et al. (31)	Norway	Total: 63 Intervention: 33 Control: 30	40.7	Female: 50% Male: 50%	Eight 90 minute session single-family intervention to improve individual and family functioning	2.5 hour psychoeducation-al group session	Hospital and patients' homes	Mental health subscales on the 36-item Short-form Health survey, Caregiver Burden Scale (ZBI), Family Adaptability and Cohesion Evaluation Scale 4th edition and Quality of Life after Brain Injury Questionnaire
Rivera et al. (28)	United States	Total: 67 Intervention: 33 Control: 34	51.1	Female: 94% Male: 6%	Problem solving training in 4 in-home sessions and 8 telephone follow-up calls	Written educational materials and telephone calls at set intervals	Patients' homes	Center for Epidemiologic Studies Depression Scale, Satisfaction With Life Scale, Pennebaker Inventory for Limbic Languidness, difficulty subscale of Caregiver Burden Scale, 52-item Social Problem-Solving Inventory Revised
Sinnakaruppan et al. (30)	UK	Total: 42 Intervention: 23 Control: 19	Not specified	Female: 78.6% Male: 21.4%	8 sessions of Educational Training Programme for memory, executive functions and emotion, especially anxiety, depression and anger	No educational input	Community	The Hospital Anxiety and Depression Scale, The General Health Questionnaire−28, The Rosenberg Self-Esteem Scale, Coping with Problems Experienced Questionnaire
Vranda and Reddy (17)	India	Total: 90 families Intervention: 50 families Control: 40 families	18–37	Female: 29%, Male: 71%	8 sessions of psychoeducation and 5–6 sessions of group intervention programme with occupational rehabilitation, speech therapy, and orthopedic consultations and financial assistance	Not specified	Hospital and community	Family Interaction Patterns Scale: leadership, communication, role, reinforcement, cohesiveness, and social support system

Authorship, the country where the study was conducted and the number of participants in the intervention and control group were included in the characteristics of the studies. The characteristics of participants were also recorded, such as whether they were civilians/ veterans, mean age and the percentage of each gender. In addition to the above data, the mode of delivery of the intervention, intervention programme, control, setting, duration and frequency of intervention, the timepoint which the intervention was conducted during the patient's journey were documented.

Caregiver burden was selected as the primary outcome, which consisted of either the 22 item and 12 item Zarit Burden Interview (ZBI), modified version of the Caregiver Appraisal Scale, Caregiver Burden Scale, Self-Perceived Burden Scale and the Bakas Caregiving Outcomes Scale. The secondary outcome measured psychological and somatic symptoms of caregivers. Four categories of other outcome measures were reported as well, namely overall health and quality of life (QoL), family functioning, TBI patients' assistance level and others. Although follow up data was frequently reported in most studies, only data collected immediately after the intervention was analyzed.

### 2.5 Risk of bias

Version 2 of the Cochrane risk-of-bias tool for randomized trials (RoB-2) was used to assess the quality of the RCTs (25). RoB-2 documents 5 domains of bias, namely bias from the randomization process, deviations from the intended interventions, missing data, measurement of outcomes and selection of the reported results. The two reviewers (CYT and ZA) screened the RCTs independently and rated each domain for low, unclear or high risk of bias across each study. The quality of all studies was then assessed to give an overall risk of bias. Any discrepancies between the assessors were discussed to reach a mutual consensus.

### 2.6 Statistical analysis

The mean and standard deviation (SD) of ZBI and BSI-18 was extracted from the results of the studies as there was sufficient homogeneity between at least three studies for both assessments. As two studies reported the mean and SD of the average score of ZBI, the data was calculated by the authors to extract the mean and SD of the total score of ZBI. A meta-analysis was conducted using RevMan 4.0 software (Cochrane Collaboration, London, UK) employing a random effects model. The mean difference and the 95% confidence interval were reported with I^2^, Chi^2^, and Tau^2^ to indicate the heterogeneity across studies. Forest plots were also created to show whether the results favored the intervention or the control group. As studies used a variety of outcome measures, outcomes other than ZBI and BSI-18 were analyzed using a narrative synthesis of the data.

## 3 Results

### 3.1 Search results

**Figure 1:**
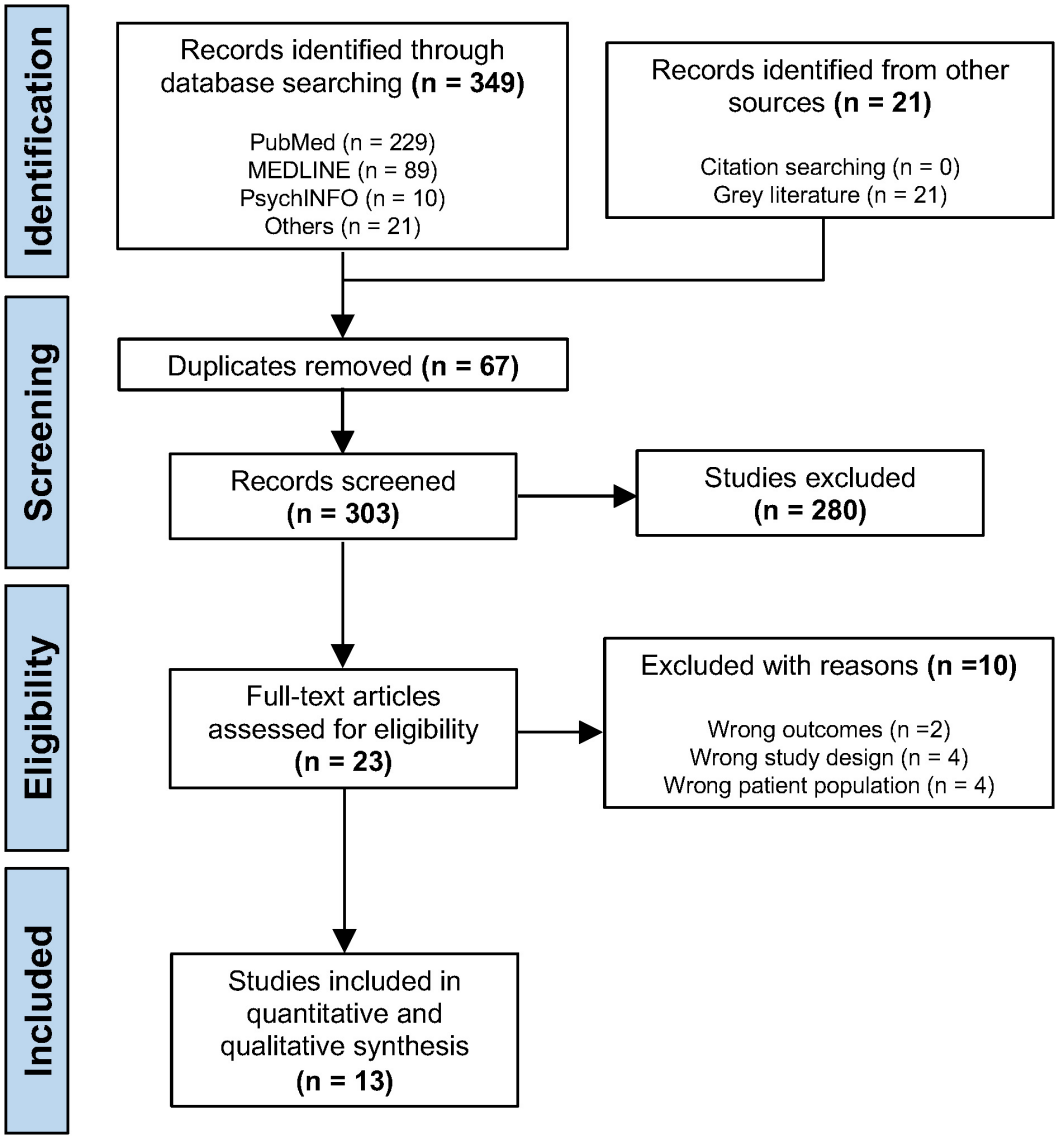
PRISMA flow diagram.

The initial search yielded a total of 370 papers, comprising 349 results from three databases and 21 references from gray literature. 67 references were identified as duplicates by Covidence (https://www.covidence.org/; Melbourne, Victoria, Australia) and were removed. 303 studies were screened according to title and abstract by the primary reviewer (CT), resulting in the exclusion of 280 studies. The remaining 23 full text studies were assessed for eligibility in the second stage of screening. Out of the 10 studies excluded, two were due to having wrong outcomes, four due to wrong study design and four for including the wrong patient population. Ultimately, 13 studies were included for the meta-analysis (Table 2). The entire process was reviewed by the co-author (ZA), including performing independent searches in the same database using the same search strings and the searches and included studies were confirmed by the co-author. The process is summarized in Figure 1 in the form of a PRISMA flowchart.

### 3.2 Study characteristics

Data extracted from the studies was summarized in Table 2. RCTs were selected to ensure potentially high methodological quality across the studies. The 13 studies were published from 2005 to 2025, encompassing a total of 1,252 participants and 90 families. 629 participants and 50 families took part in the interventions while 623 participants and 40 families took part in the control groups. The majority of studies were conducted in Western countries, with eight studies based in North and South America (9, 11, 14, 15, 18, 26–28), three in Europe (29–31) and two in Asia (10, 17). Caregiver age mostly ranged from 36–52, with only 1 study having younger participants (aged 18–37) (17). Most of the studies had >75 % participants as females (9–11, 14, 15, 18, 27, 28, 30) while three studies had female percentages lower than 50% (17, 26, 31). Most caregivers were at the age of 36–50, except for 1 study (17). The time of injury of TBI patients ranged from acute post-injury to 10 years. One study specifically focused on veterans with TBI while others recorded interventions for civilians.

Interventions differed considerably in program structure, duration and content. The interventions lasted from 10 days to 2 years. Intervention methods involved face to face interactions, remote programs based on web or telephone communication or a combination of both. Six studies had their intervention delivered face to face (9, 17, 18, 26, 30, 31), four had blended approaches (10, 27–29), and three were conducted using remote interventions only (11, 14, 15). The types of remote interventions included mobile health apps with online chat functions, telephone follow up, interactive websites. In contrast, in-person interventions encompassed psychoeducational sessions delivered by medical staff, problem solving training, group intervention programs, individual peer mentoring and home visits. All face to face interventions were conducted in hospitals or in patients' homes. In-person interventions were conducted weekly, or biweekly or once a month, usually spacing out during the end of the treatment. On the other hand, remote interventions tend to allow caregivers higher flexibility in using the medical applications, generally at their own pace. Control groups were either given standard care at the local hospital or written materials without additional explanation from medical staff.

### 3.3 Risk of bias assessment

**Figure 2:**
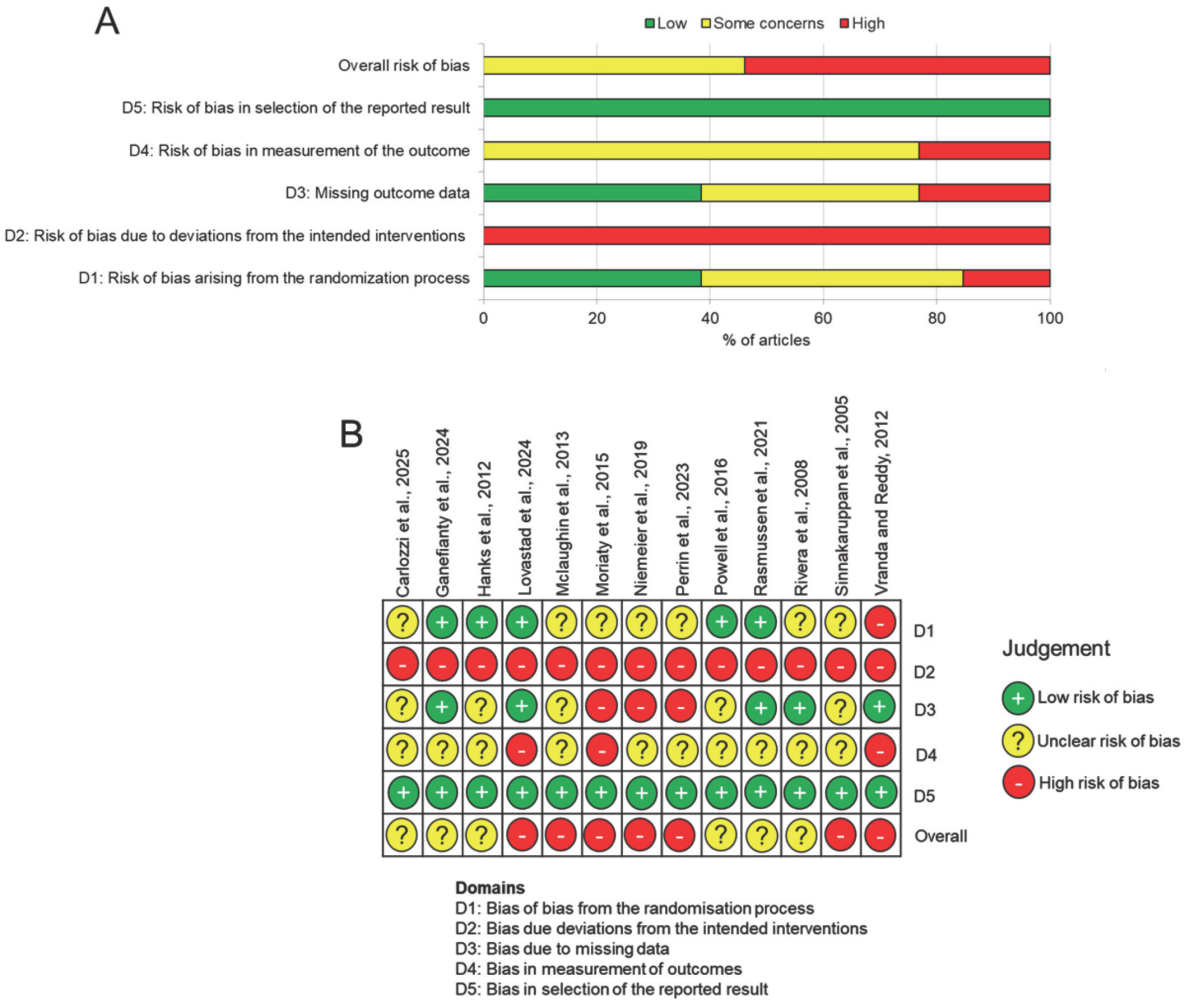
Risk of bias assessment. **(A)** Summary chart for risk of bias in all studies. **(B)** Risk of bias in individual studies for each domain.

The risk of bias-2 tool (RoB-2) was used to assess the quality of the included RCT studies (Figure 2). Most studies had an overall moderate to high level of bias, with seven studies rated as high (9, 15, 17, 18, 27, 29, 30) while six studies had some concerns (10, 11, 14, 26, 28, 31). Due to the nature of the interventions, all studies were not able to blind the participants to their allocated interventions. The staff implementing the programs were also aware of the caregivers' allocated interventions as blinding could not be conducted. Thus all studies had a high risk of bias due to deviations from the intended interventions (D2). Two studies were rated high in bias from the randomization process (D1) as the details of the randomization processes were not specified (17, 18). Six studies had an unclear risk of bias as the randomization process was not described in detail (9, 14, 15, 27, 28, 30). There were three studies rated as high for bias due to missing data (D3) as they had >25% of missing data (9, 18, 27) while five studies raised some concerns due to a 10%−25% attrition rate (11, 14, 15, 26, 30). Most dropouts were lost to follow-up or caregiver withdrawal, raising concerns on the sustained participation and effectiveness of the interventions. There were also concerns related to bias in measurement of outcomes (D4) as most outcome measures were subjectively rated by caregivers themselves. Given the subjective nature of the primary outcome, the potential for measurement bias was notable. Objective measures were typically included as secondary outcomes, such as psychological metrics and vital signs, knowledge in caregiving and health measures for TBI patients. Finally, bias in selection of the reported result (D5) was mostly rated as low to moderate risk of bias without evidence of selective reporting amongst studies.

### 3.4 Composition of the intervention providers

In considering the composition of the team that provided the intervention to the carer's, three studies did not clearly state the qualifications of the interventionists (14, 17, 28), whilst one study was a web-based intervention developed by the Brain Injury Partners (15) (Table 3). However, many studies had one specialist that included a nurse, social worker, psychologist or researcher, occupational therapist of a neuropsychologist (10, 11, 18, 27, 30, 31). Only two studies reported that a multidisciplinary team delivered the interventions, including medical doctors, nurses, community worker, physiotherapist and a neuropsychologist (26, 29). One study reported that a team of three psychologists delivered the intervention (9).

**Table 3:** Composition of the intervention provider.

**Authors**	**Composition of the intervention provider**
Carlozzi et al. (14)	Not clearly stated but authorship includes researchers, doctors, psychiatrists and biostatisticians
Ganefianty et al. (10)	Nurses
Hanks et al. (26)	Psychologist, nurse and a community outreach worker
Løvstad et al. (29)	A medical doctor, a psychologist, a physiotherapist, and a neuropsychologist
McLaughlin et al. (15)	Web-based intervention
Moriaty et al. (27)	Occupational therapist
Niemeier et al. (9)	Psychologists (x3)
Perrin et al. (18)	Psychologists of research assistants
Powell et al. (11)	Social worker
Rasmussen et al. (31)	Physical therapist
Rivera et al. (28)	Interventionist
Sinnakaruppan et al. (30)	Qualified neuropsychologist
Vranda and Reddy (17)	Composition of interventionists not reported

### 3.5 Outcomes

#### 3.5.1 Primary outcome data: caregiver burden

The primary outcome measures (caregiver burden) were mostly self-reported. Five studies utilized the ZBI (9, 10, 18, 29, 31), one study reported a subscale of the ZBI (28) and two studies used the modified version of Caregiver Appraisal Scale (14, 27). In the five studies, the term “Caregiver Burden Scale” is used interchangeably with “The Zarit Burden Interview”. The ZBI is a self-reported assessment that measures the subjective burden in caregiver experiences, indicating the strain on personal, social and financial aspects of life (32). Caregivers are required to answer 22 items on a 5-point Likert scale of 0–4, where lower scores indicate a lower caregiver burden.

**Figure 3:**
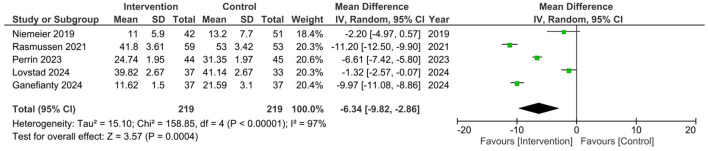
Meta-analysis of primary outcome data for ZBI scores in appropriate studies (9, 10, 18, 29, 31).

A meta-analysis for studies that reported the ZBI results showed a mean difference of 6.34 favoring the intervention group (*p* < 0.00001), but with high heterogeneity displayed (*I*^2^ = 97%, Chi^2^ = 158.85). In addition, a high degree of between study variability in effect sizes across studies was reflected. Two out of the five studies reported statistically significant outcomes (10, 18), while the results of the other three studies were non-significant (9, 29, 31). However, an overall positive effect favoring the intervention was detected (Figure 3).

#### 3.5.2 Secondary outcome data: psychological symptoms

For psychological symptoms, three studies utilized the BSI-18 (9, 11, 26), two studies used the Center for Epidemiologic Studies Depression Scale (CESD) (27, 28), another two used the Patient Health Questionnaire (PHQ-9) (18, 29) while three others used other assessments (14, 30, 31). The BSI-18 is a 18-item self-reported checklist that assesses the psychological status in patient populations. The global severity index of the BSI-18 reports the results on all 18 items, which could be further divided into three 6 items scales, measuring somatisation, anxiety and depression.

**Figure 4:**
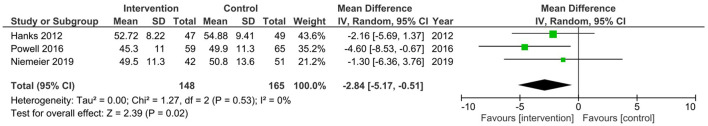
Meta-analysis of secondary outcome data for BSI-18 scores in appropriate studies (9, 11, 26).

The global severity index of BSI-18 was used to perform a meta-analysis. A mean difference of 2.84 favoring the intervention group was detected, indicating a statistically significant effect (*p* = 0.02). Low heterogeneity among studies was found (*I*^2^ = 0%, Chi^2^ = 1.27), demonstrating consistent intervention effects in different programs. Although a fixed effect model was appropriate for the statistical analysis, a random effects model was still used as a conservative approach to detect heterogeneity across studies. Two studies reported non-significant results (9, 26) while one study had positive effects on caregivers' mental health (11). However, an overall positive effect favoring the intervention was detected (Figure 4).

Only one study stated that the primary outcome of caregiver burden in male carers was associated with an increased risk of negative outcomes after the intervention (14). However, none of the other studies reported any differences in outcomes when carers were male as opposed to female (9–11, 15, 17, 18, 26–31).

## 4 Discussion

The primary objective of this study was to determine whether caregiver interventions are effective in reducing caregiver burden and psychological distress. We identified 13 studies that met our inclusion/exclusion criteria, encompassing a total of 1,252 participants and 90 families. Interventions differed considerably in structure, duration and content and lasted for 10 days to 2 years. The risk of bias was judged as moderate to high since some studies were not able to blind allocation of participants to groups due to the nature of the study. The primary outcome measure for caregiver burden was either the Zarit Burden Interview (ZBI) or the Caregiver Burden Scale and a meta-analysis from pooled data from some of the studies evaluated using the ZBI favored the intervention group. The global severity index of BSI-18 was also significant and favored the intervention. These results suggest that caregiver burden is significant and intervention helped reduce this burden significantly. Due to the high risk of bias, however, more high quality studies are required to make definitive conclusions.

Based on the findings of this study, it can be concluded that TBI caregiver interventions are effective in reducing caregiver burden. Nevertheless, the results should be interpreted with caution. The high heterogeneity observed in ZBI results likely reflects real world variations due to differences in population characteristics and intervention types, which enhances the generalizability of the results across diverse settings. Four out of eight studies reported significant reduction in caregiver burden. Of the four studies that reported a significant reduction in caregiver burden, three incorporated psychoeducation and skills training, suggesting that improvements in practical caregiving abilities and knowledge may be essential components of effective interventions (10, 18, 27, 31). Both face-to-face and blended delivery modes appeared effective in reducing caregiver burden. All four studies were conducted in hospital and home settings, with intervention durations ranging from 1 to 4 months. Three studies involved weekly or biweekly contact with caregivers (18, 27, 31) and three studies were implemented during the transition from hospital to patients' homes within 1 year of injury (10, 18, 31). These findings, paired with the meta-analysis result for ZBI, indicate caregiver interventions may lessen caregiver burden significantly. However, the inconsistency of results from the studies complicates the interpretations as overall methodological limitations and small sample sizes reduces certainty of these results. Therefore, while interventions may be beneficial, the variability in study quality requires careful consideration and further investigation for the application of interventions.

Findings of the secondary outcome are consistent with current literature, which reports small improvements in psychological symptoms following caregiver interventions. The low heterogeneity of results reflects statistical consistency, increasing the confidence in the robustness of the findings. Despite variations in delivery methods, the positive effects of interventions were consistent across different contexts. This suggests that the observed benefits of multi-component TBI caregiver interventions were stable. Given that all 3 studies were done in the United States in the meta-analysis, the homogeneity might partly reflect the similarity of study contexts, which limits generalisability to lower or middle income countries. Eight out of ten RCTs in this study reported improvements in depression and anxiety scores in various outcome measures, which matched with the findings in BSI-18. The interventions lasted from 1 month to 2 years while contacts were arranged at least once a month. All interventions that improved depression and overall mental health outcomes included an emotional support component, such as follow-up contact with medical staff or educational sessions on emotional regulation that facilitated sharing. Blended and face to face interventions worked most effectively in alleviating psychological distress. Interventions that support caregivers during the transition from hospital to home tend to lead to better psychological outcomes as well. As many caregivers feel unprepared for this change, convenient and easily accessible programs can alleviate anxiety and ease the transition. The results suggest that home visits and telephone follow-ups facilitate a smoother transition and enable timely resolution of problems as they arise. This highlights the importance of providing both psychological and practical support to improve caregivers' wellbeing. However, due to the small number and the modest quality of studies, the results should be considered alongside the broader body of literature before making definitive practice recommendations.

Placing the results of this study in wider literature, comparison with the previous systematic review must be considered, as notable advancements in intervention studies have been observed (20). The findings suggest that intervention approaches applied predominantly in Western countries may also be effective in Asian contexts when adapted to place greater emphasis on social and emotional support. Cultural contexts appeared to influence intervention priorities, where studies from Asian countries more frequently emphasized family functioning and support, whereas studies from Western countries tended to focus on individual-level outcomes, reflecting differing societal values. The differences in content also lead to the variety of outcome measures. Compared to previous systematic reviews, outcome measures have become more standardized, allowing a meta-analysis to be done, yet they have also grown more diverse. Family functioning outcome measures have become increasingly common in current intervention studies, reflecting that support should extend beyond the individual caregiver to the family unit as a whole. Nevertheless, current literature still contains relatively few outcome measures specifically validated for TBI caregivers. One such tool, the Traumatic Brain Injury Caregiver Quality of Life (TBI-CareQOL), was validated in 2020 but it has yet to be widely adopted in interventions studies (33).

In terms of assessing how easy the interventions were for both participants and providers, this was difficult as interventions were delivered in a variety of way, ranging from web-based, app-based, face-to-face, online chats, home visits and telephone calls. However, interventions can be delivered in a variety of settings, including in patient's own homes which may be easier to follow without the need for repeat in-person visits. However, this relies on patients making sure that they adhere to the intervention plan, which may be hard to follow. Future research should assess how best to deliver these interventions and whether remote or web-based platforms may offer improved adherence due to their flexibility.

Several limitations should be considered when interpreting the findings of this review. Although a comprehensive multi-database search was conducted, relevant studies may have been missed due to publication bias and language restrictions as those without English full text manuscripts were excluded. Second, there is moderate to high risk of bias in the studies, with common issues including high attrition rate, inadequate blinding and incomplete reporting of randomization processes. Third, the predominance of studies conducted in high-income countries, where healthcare systems and caregiver support services may differ substantially from those in low and middle income countries. This limits the generalisability of findings to global contexts. Cultural differences in caregiving roles, family values and coping mechanisms may also influence the acceptability and effectiveness of interventions. Thus, interventions focusing on the needs of TBI caregivers with different ethnicities should also be developed to generalize interventions to other populations. Finally, as there are no standardized outcome measures for TBI caregivers specifically, conclusions should be drawn cautiously and considered alongside the broader literature. Future updates to this review should aim to incorporate these developments, standardize outcome measures and assess the long-term effects of caregiver programs.

Another limitation is that across the 13 studies, there was a variety of specialists, ranging from nurses to psychologists, that delivered the intervention and so there is considerable methodological heterogeneity and comparisons of potential caregiver burden improvements across the included studies must be interpreted with caution. Future studies should look to standardize the composition of the team that delivers the intervention, with perhaps first identifying which specialist or a multidisciplinary team of specialist might provide the best training for the intervention.

## 5 Conclusion

This systematic review found that multi-component interventions are beneficial in reducing caregiver burden and psychological distress among TBI caregivers. Interventions with both practical and psychological support, implemented at least once a month should be considered during the TBI patient's transition from hospital to community. Sessions using a blended or face to face approach are encouraged to facilitate caregivers in meeting the needs of their loved one. However, while benefits were observed over various populations and delivery modes, results should be interpreted with caution due to methodological limitations, which are consistent with previous systematic reviews. The heterogeneity of studies makes it difficult to develop definitive clinical guidelines as differences in intervention design, delivery and outcome measures limit the ability to establish universally applicable recommendations. Future research should prioritize rigorous study designs and the validation of standardized outcome measures in TBI caregiving to enable clearer comparisons between interventions. Overall, well-structured caregiver interventions appear promising for integration into hospital standard care and rehabilitation pathways, but higher quality research is required to confirm their effectiveness, determine the best specialist or teams of specialists to deliver the intervention and thus guide broader implementation.

## Data Availability

The original contributions presented in the study are included in the article/supplementary material, further inquiries can be directed to the corresponding author.
